# Enhancement of oil forensic methodology through the addition of polycyclic aromatic nitrogen heterocycle biomarkers for diagnostic ratios

**DOI:** 10.1007/s10661-023-10941-3

**Published:** 2023-02-20

**Authors:** Paige McCallum, Taylor Filewood, Julia Sawitsky, Honoria Kwok, Pamela Brunswick, Jeffrey Yan, Leah Chibwe, Krishnaja Tikkisetty, Dayue Shang

**Affiliations:** 1 Science and Technology Branch, Pacific Environmental Science Centre, Environment and Climate Change Canada, Pacific and Yukon Laboratory for Environmental Testing, North Vancouver, B.C Canada; 2 Institute for Environmental Change and Society, University of Regina, SK Regina, Canada

**Keywords:** Oil spill forensics, Polycyclic aromatic nitrogen heterocycles, Biomarkers, Gas chromatography quadrupole time-of-flight mass spectrometry

## Abstract

Current oil spill forensic identification of source oils relies upon hydrocarbon biomarkers resistant to weathering. This international technique was developed by the European Committee for Standardization (CEN), under EN 15522–2 Oil Spill Identification guidelines. The number of biomarkers have expanded at pace with technological advances, while distinguishing new biomarkers becomes more challenging due to interference of isobaric compounds, matrix effects, and high cost of weathering experiments. Application of high-resolution mass spectrometry enabled exploration of potential polycyclic aromatic nitrogen heterocycle (PANH) oil biomarkers. The instrumentation showed reduction in isobaric and matrix interferences, allowing for identification of low-level PANH and alkylated PANHs (APANHs). Weathered oil samples, obtained from a marine microcosm weathering experiment, enabled comparison with source oils to identify new, stable forensic biomarkers. This study highlighted eight new APANH diagnostic ratios that expanded the biomarker suite, increasing the confidence for identifying highly weathered oils back to their source oil.

**Supplementary Information:**

The online version contains supplementary material available at 10.1007/s10661-023-10941-3.

## Introduction

Despite intensive research, public awareness, and preventive measures, oil spills are still a common occurrence. Oil finds its way into the environment through both natural and anthropogenic causes, including by leakage from pipelines and shipwrecks (Oil Spills, 2022; Wang & Stout, 2007). While the number of oil spills over the past five decades has decreased, there have been 39 global oil spills, 10 of which have been reported as greater than 700 tonnes since 2015 (Roser & Ritchie, 2022). Even a relatively small amount of oil could result in serious impacts to susceptible ecosystems, due to the high toxicity level of components and persistence of toxic compounds (Oil Spills, 2022). The oil spilt during the Deep Water Horizon disaster of 2010 is one such case where the persistence of harmful oil–derived hydrocarbons continues to affect the surrounding ecosystems (Deepwater Horizon's Impact on Wildlife, 2022).

Following an oil spill, a natural process known as weathering occurs which affects all compounds within the oil to varying degrees (Reyes et al., 2014). The types of weathering that can occur are outlined in Table 1. In addition to environmental exposure conditions, some of the oil compounds are more resistant to weathering in comparison to others (Reyes et al., 2014). Consequently, weathered oil components can vary significantly, creating difficulties in oil spill to source identification and matching. Overcoming these issues has been the focus of a variety of analytical techniques (Lundberg, 2019; Reyes et al., 2014; Yang et al., 2015).

**Table 1:** Weathering of oil in marine environment and summary of the compound groups most affected (Filewood et al., 2022a)

**Weathering process**	**Description**	**Compounds most affected**
Photo oxidization	UV-sensitive compounds undergo degradation. Affected by oil film thickness and the presence of unsaturated and aromatic compounds	APAHs, naphthalene (and alkylated derivatives), unsaturated FAMEs
Evaporation	Low-boiling-point compound transfer into the atmosphere. Occurs shortly after a spill and the removal of such compounds increases the concentration of other biomarkers in spilled oil	Alkanes (low molecular weight), alkylated benzene
Dissolution	Smaller aromatic oil compounds incorporating into aqueous media post spill. Affected by wave action and other natural conditions	Low ring aromatic hydrocarbons (preferentially less substituted)
Biodegradation	The decomposition of oil compounds by microorganisms in environmental media. Affects mainly saturated and alkylated hydrocarbons	Alkanes (all), alkylated benzene, alkylated toluene, FAMEs, APAHs, PAHs

With respect to oil spill forensics, polycyclic aromatic hydrocarbons (PAH) have been studied intensively because of their known toxicity, carcinogenic properties, and high concentration within various petroleum oils (Anyanwu & Semple, 2015; Ghosh & Mukherji, 2021). However, despite their structural similarities (seen in Table 2) and presence in oils, the nitrogen-containing polycyclic aromatic nitrogen heterocycles (PANH) and their alkylated forms (APANH) have been excluded from analysis in these studies. Reasons for this omission are likely due to the high cost of weathering experiments, severe matrix effects, and interference by isobaric compounds (Najeeb & Kadhane, 2017; Witter & Nguyen, 2016). Based on the structural similarities, it is anticipated that PANHs and APANHs would weather in similar ways to the classic PAHs and APAHs, suggesting their potential as biomarkers.

**Table 2:** List of APANH potential biomarkers and closest classic PAH structures

Target APANH	Parent PAH
C1-Benzo[a]carbazole	Chrysene
C2-Benzo[a]carbazole	Chrysene
C3-Benzo[a]carbazole	Chrysene
C4-Benzo[a]carbazole	Chrysene
C2-Benzo[c]acridines	Benzo(a)anthracene
C3-Benzo[c]acridines	Benzo(a)anthracene

Currently, an internationally recognized oil source identification method, developed by the European Committee for Standardization (CEN), using the EN 15522–2 Oil Spill Identification guidelines, is used to identify source oils from weathered samples (CEN, 2021). The CEN method has been adopted by an increasing number of environmental and analytical labs worldwide and relies on gas chromatography with mass spectrometry detection to analyze for a range of biomarker compounds (CEN, 2021). These biomarkers, differing in resistance to weathering, are used in response ratios to one another, relying on the assumption that compounds in the same group are likely to weather at similar rates. As a result, the determined ratio for a spilled oil is generally more comparable with its corresponding source sample than with other oils (Malmborg et al., 2020; Yang et al., 2008).

The biomarker ratios used in the current CEN method include many well-established oil components such as alkanes, terpanes, steranes, sesquiterpanes, phenanthrenes, dibenzothiophenes, benzonaphthothiophenes, fluorenes, and chrysenes. The inclusion of heterocyclic PAHs as biomarkers is not common, although a recent study by Filewood et al. (2022a) demonstrated the success of including 19 new polycyclic aromatic sulfur heterocycle (PASH) and alkylated polycyclic aromatic sulfur heterocycle (APASH) biomarker ratios in the forensic diagnostics. This discovery prompted us to investigate the potential of exploring another group of heterocyclic PAHs, i.e., PANH and APANH, as biomarkers and their diagnostic ratios in oil forensics. The varying levels of these non-classical biomarkers in different oils add to the fingerprint of the oil. Experienced oil diagnostic forensic analysts recognize the benefits of including additional biomarkers, particularly in circumstances of extreme weathering where oil spill source identification can be challenging. Therefore, increasing the number of diagnostic ratios to include additional stable heterocyclic PAHs beyond those of the classic biomarker ratios can increase confidence in the diagnostic outcome.

The goal of the current study was to assess the ability to detect trace levels of PANH and APANH compounds, evaluate their stability during weathering, and finally assess their use in biomarker diagnostic ratio analysis. Microcosm studies were set up using weather conditions experienced in the Pacific Northwest marine environment to analyze the effects on heavy and crude oils. The study aimed to fill a current gap in the classic diagnostic ratio analysis methodology by identifying and screening for low-level PANH. This study took advantage of the high specificity of the gas chromatography quadrupole time-of-flight (GC-QToF) mass spectrometry to asses PANH and APANH levels in six petroleum oils before and after artificial weathering. This novel study aimed to enhance the current oil spill forensic tools and allow for higher confidence when identifying the source oil when a spill occurs in the environment.

## Materials and methods

For details regarding purchased reagents, unweathered extraction and sample preparation, microcosm and weathering conditions, weathered oil extraction and sample preparation, and GC-QToF procedure and data collection, see Supplementary Information (SI).

### Petroleum oils

Table 3 lists the six petroleum oils used in this study with complete information provided in the SI Table S1.

**Table 3:** Six crude oils used in this study, including abbreviated names

**Name**	**Abbreviated name**
Transport Canada – Oceanic HFO filter 09.02.30	HFO TC
Plains midstream Red Deer River, 08Jun2012	PM RDR
Alberta Sweet Mix Blend	ASMB 5
Hibernia	HB
HFO 6303, ESTS 20051031–0601.2 BPH 22Nov2012	HFO 6303
MC-252 ESTS 20100916–1622.6 BPH 22Nov2012	MC 252

### GC-QToF procedure and data collection

Detailed explanation on the data processing was presented in previous work by Kwok et al. and Park et al. (Kwok et al., 2019; Park et al., 2018). Due to their similarities, the PANHs and the APANHs were processed using the same technique as for PAHs and APAHs. Agilent’s MassHunter Qualitative analysis was used to determine the retention times for the PANHs and APANHs target ions (SI Table S3). Once determined, Agilent’s MassHunter Quantitative analysis was used to determine the height and area with adjustment of the mass extraction window to include the PANH and APANH target ions (Filewood et al., 2022a; Kwok et al., 2019; Park et al., 2018).

### Diagnostic ratio analysis

In this study, diagnostic ratio analysis was based on the processing described by Filewood et al. (2022a) together with the classic CEN EN 15522–2 Oil Spill Identification guidelines (CEN, 2021). An Excel^®^ spreadsheet was created to compare the combined 78 classic biomarker ratios plus the additional PASH and APASH ratios. In addition, observed PANHs were screened for their potential as biomarkers in ratio to other biomarkers. The final selected PANH and APANH biomarker ratios were then included in the spreadsheet for reporting.

Agilent’s MassHunter software was used to complete peak integration of each PANH and APANH compound that had detectable peaks. This was completed for the unweathered and weathered oils to determine which height and area peaks remained detectable following weathering. Through this careful analysis, eight new ratios based on peak areas were added to the final Excel sheet (Sect. 3.3). The relative percent difference between the weathered and source oil ratios was compared, with a final acceptance as a match being < 59 out of 105 diagnostic ratios flagged as greater than a 14% relative difference (14% is internationally accepted as the percent difference criterion) (CEN, 2021).

## Results and discussion

### Preliminary analysis by classic CEN method

In the current study, the artificially weathered oil samples were used as the “spilled oil” and the corresponding unweathered samples were regarded as the “source oil.” The unweathered oils were analyzed prior to weathering with minimal sample loss or change, and the results were taken as a baseline in this study. One goal of this study was to identify new diagnostic ratios that can withstand Canadian West Coast weathering and reduce the number of flagged ratios present in the current classic CEN method. Flagged ratios were determined if the weathered to unweathered ratios had a percent difference greater than 14% (CEN, 2021). The weathered to unweathered oil differences in biomarker ratios were presented both numerically and visually in order to better observe if the spill to source comparison was a likely match. For a spill and source oil to be considered a probable match, there should be a high number of ratios unflagged in comparison to other oil samples. In this study, the original (classic) 78 ratios used in the CEN method were first compared (CEN, 2021). Based on the current findings (Table 4), a good match for severely weathered oils may be defined by having more than 34 unflagged out of the total 78 ratios (italicized). When comparing a spill sample with its corresponding source, more unflagged ratios increased the confidence in identification of an environmental sample to the source material (Table 4 in italics).

**Table 4:** Number of acceptable (unflagged; highlighted in italics) diagnostic ratios when comparing the weathered to unweathered oil samples by the classic CEN method

Using 78 classic biomarker ratios	HFO TC	PMRD	ASMB	HB	HFO 6303	MC
Weathered HFO TC	*38*	15	19	11	18	19
Weathered PMRD	17	*40*	20	8	15	21
Weathered ASMB	16	15	*42*	11	18	22
Weathered HB	6	7	10	*34*	10	14
Weathered HFO 6303	17	14	20	14	*44*	25
Weathered MC	15	23	21	11	20	*42*

**Fig. 1:**
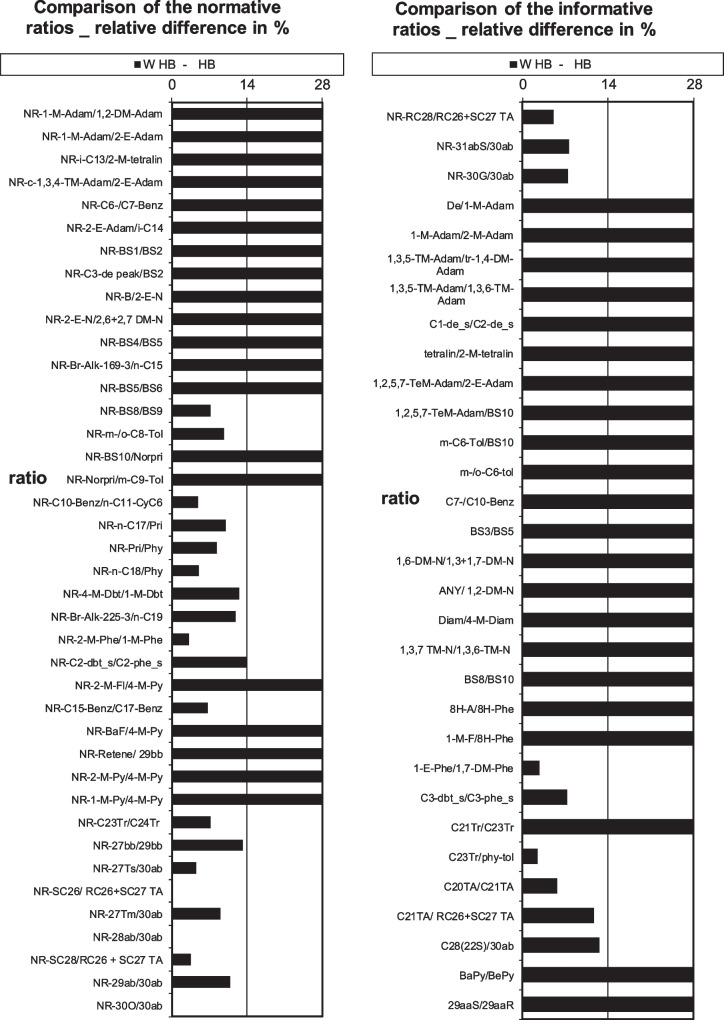
Visual representation of classic diagnostic ratio (78) results for the HB weathered versus corresponding source oil (FAME excluded for clarity of figure)

The current result can be observed visually in Fig. 1 for the classic CEN methodology comparing the 78 classic diagnostic ratios. It is shown that quite a few of the ratios are calculated to be above 14% difference in the comparison between the weathered and unweathered HB oil. This result will be used as a baseline to confirm the enhancement of the CEN methodologies confidence in determining a match using additional PANH biomarkers.

**Fig. 2:**
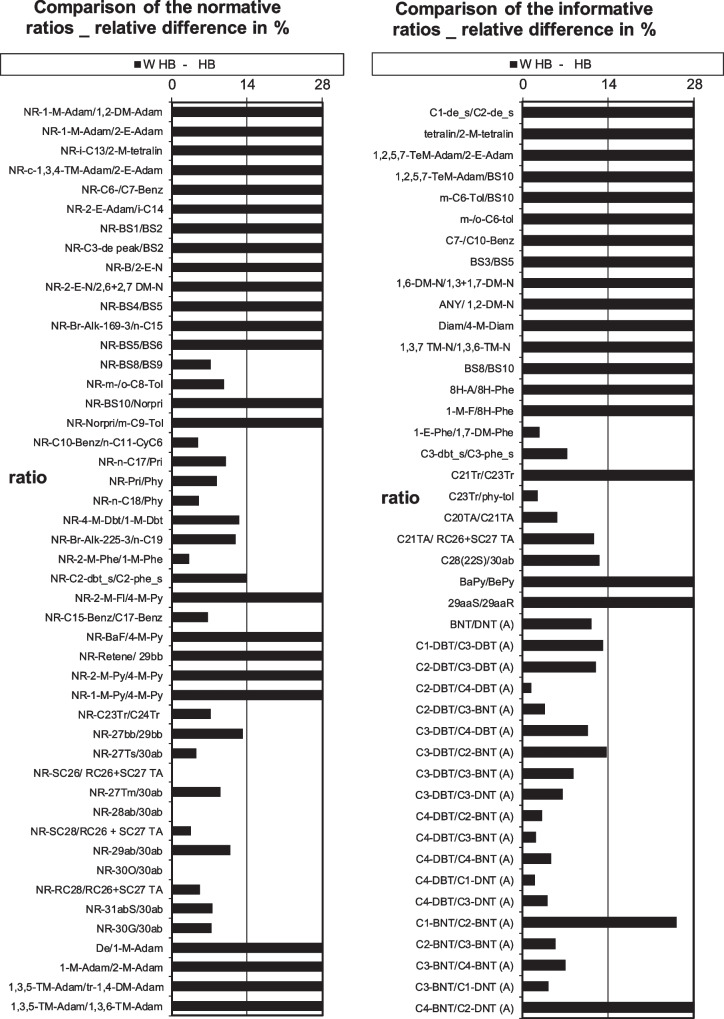
Visual representation of classic diagnostic ratio (78) results and PASH/APASH diagnostic ratio (+ 19) results included (right bottom) for the HB weathered versus corresponding source oil (FAME excluded for clarity of figure)

In support of the findings of Filewood et al. (2022a), the additional inclusion of PASH to the CEN diagnostic ratio analysis clearly enhanced the number of acceptable unflagged ratios (Table 5). This result is visually demonstrated in Fig. 2. It can be seen that the 19 additional PASH ratios (bottom right of figure) increase the proportion of ratios with differences below 14% when comparing the weathered and unweathered HB oil.

**Table 5:** Number of acceptable (unflagged; highlighted in italics) diagnostic ratios when comparing the weathered to unweathered oil samples by the classic CEN method and additional PASH/APASH ratios

Using 78 classic biomarker ratios and addition PASH/APASH ratios	HFO TC	PMRD	ASMB	HB	HFO 6303	MC
Weathered HFO TC	*57*	21	23	16	25	23
Weathered PMRD	24	*59*	26	19	19	28
Weathered ASMB	20	22	*58*	18	19	28
Weathered HB	10	11	15	*51*	11	25
Weathered HFO 6303	23	17	23	17	*63*	28
Weathered MC	17	21	26	23	21	*61*

**Table 6:** List of the determined PANH Biomarkers

**Compound name**	**Determined to be biomarkers**
C1-Benzo[a]carbazole	Yes
C2-Benzo[a]carbazole	Yes
C3-Benzo[a]carbazole	Yes
C4-Benzo[a]carbazole	Yes
C2-Benzo[c]acridines	Yes
C3-Benzo[c]acridines	Yes

### Determining additional PANH and APANH biomarkers

To enhance the current CEN plus PASH methodology for application of severe weathering spill source identification, additional PANH and APANH biomarkers were assessed for inclusion (SI Table S3). Biomarkers have been commonly studied for oil spill fingerprinting using gas chromatography-flame ionization detection (GC-FID) and gas chromatography-mass spectrometry (GC–MS) (Chua et al., 2020b; Filewood et al., 2022a; Kwok et al., 2019; Park et al., 2018; Shang et al., 2014; Yan et al., 2018; Yang et al., 2020). However, the routine inclusion of PANH compounds has previously been avoided due to the more challenging interferences caused by the closely eluting PAHs, PASHs, and PANHs (Yang et al., 2020).

A study completed by Zhang et al. (2018) explored the characterization of PANH compounds in petroleum and crude oils through the use of GC–MS and liquid chromatography-high resolution mass spectrometry (LC-HRMS). It found that PANH compounds with higher alkylation levels were detected in abundance in crude and heavy fuel oils. Based off the previously stated study, they determined that APANHs could be used for possible oil source identification. In the current study, APANHs were analyzed using high-resolution GC-QTOF mass spectrometry allowing for more accurate separation and identification of the various APANHs (Filewood et al., 2022c). Height and area responses were then analyzed to allow for the determination of potential biomarkers.

For a compound to be employed as a biomarker in oil spill diagnostics, one of the favorable characteristics is to show a relatively small change following weathering. The area and height responses of the PANH and APANH compounds were analyzed for their potential in this regard. The stability of the compounds was determined by examining the chromatographic area and height response following extreme weathering in outdoor microcosms. In the current study, a large number of PANHs and APANHs became undetectable. The exception was a group of APANHs listed in Table 6, i.e., C1-Benzo[a]carbazole, C2-Benzo[a]carbazole, C3-Benzo[a]carbazole, C4-Benzo[a]carbazole, C2-Benzo[c]acridines, and C3-Benzo[c]acridines. The structure of these compounds is provided in SI Fig. S1. The biomarkers all possessed four membered rings. Benzene rings with delocalized electrons above and below the plane of the ring makes it a more stable structure (Structure and Stability of Benzene, 2022). It is well recognized that the more rings a compound has, the harder it is to break down during decomposition. These findings are consistent with the degree of stability that the determined biomarkers display.

Early PANH-related research demonstrated that PANHs are derivatives of PAH, with the assumption that PANH and PAH would be affected similarly by weathering (Anyanwu et al., 2015). It was observed in this study that fewer PANH biomarkers were found to survive extreme weathering in comparison to PAHs, which is concluded to be due to the decrease in stability that the nitrogen atom infers. The findings of Levandowski et al. (Levandowski et al., 2020) stated that increasing the nitrogen substitution in a benzene ring also increases the Diels–Alder reactivity, which subsequently decreases the stability of the compound. In addition, parent PAHs often biodegrade faster than their alkyl homologues (Wang & Stout, 2007) and APAHs are found to have a higher concentration than PAHs in petroleum oils (Park et al., 2018). However, extensive weathering exposes oils to extreme heat and sunlight, inducing photo-oxidization of APAHs (Wang & Stout, 2007), a further reason for the observed low abundance of APANH found in the studied oils. Nevertheless, six APANH biomarkers were able to be determined and were explored for their ability to enhance the CEN method for oil spill identification.

### Diagnostic ratio analysis

It has been determined previously that PAHs and APAHs are affected differently by weathering (Filewood et al., 2022a; Wang & Stout, 2007). In this study, most PANH compounds were not found to be stable enough post weathering for routine diagnostic ratio analysis. As a result, only the stable APANHs were explored for their diagnostic ratio potential. Results found that only comparing APANH to APANH compounds was successful, supporting the findings of a previous study involving APASHs (Filewood et al., 2022a). To ensure the accuracy and repeatability of the results, six duplicated oil results were studied. Of the six duplicate oils, the determined biomarkers work for 100% of the oils, surpassing the criteria of 80% acceptance set by a previous study by this lab (Filewood et al., 2022a).

Both the peak areas and heights of the biomarker compounds were tested when determining the diagnostic ratio. The ratios, as seen in SI Table S4, were selected for their ability to correctly match each weathered oil to its unweathered source (< 14% relative ratio percent difference). It was found that the peak area was more suitable for diagnostic ratio determination than that of using the peak height (Table S4). The use of peak height is reportedly more suitable when a noisy baseline and compressed peaks are observed (CEN, 2021).

### APANH diagnostic ratio quality control and quality assurance

**Fig. 3:**
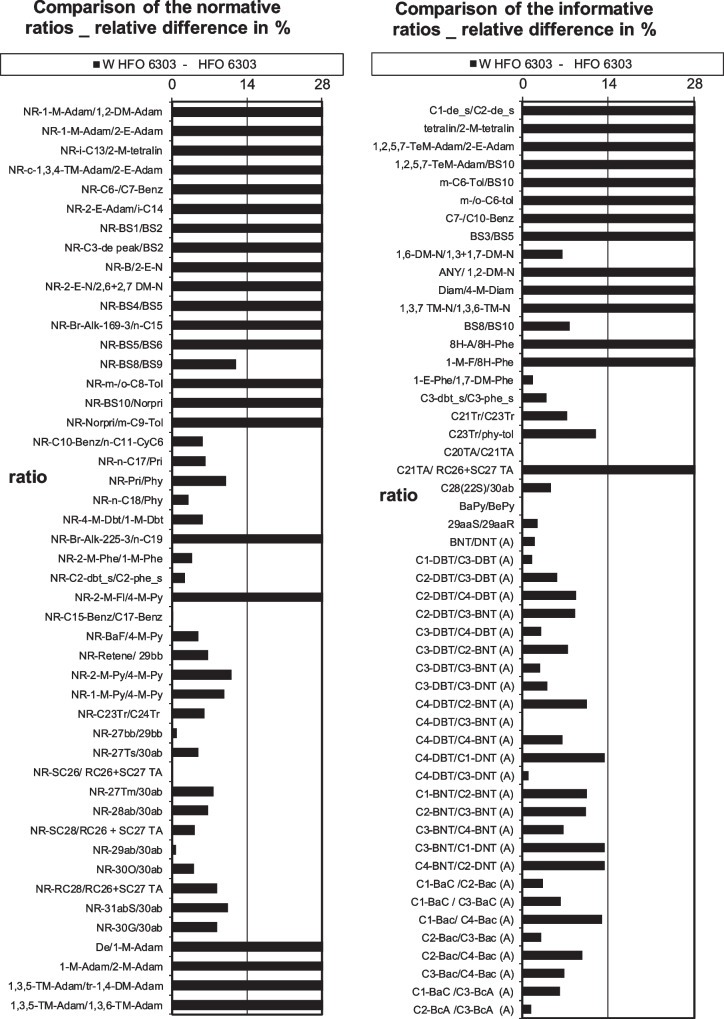
Visual representation of classic diagnostic ratio (78) results, PASH/APASH diagnostic ratio (+19) results, and APANH diagnostic ratio (+ 8) results included (right bottom) for the duplicate HFO 6303 weathered versus corresponding source oil (FAME excluded for clarity of figure)

Once the acceptance criteria for the expanded diagnostic method including the eight newly determined APANH biomarker ratios were proposed, a quality assessment was performed by application to an additional analysis on all of the oils. The visual results for HFO 6303, HB, and MC 252 of weathered versus unweathered oil samples can be seen in Fig. 3, Figs. S2, S3 respectively. The samples were run a year prior to the current study. Results from two separate analysts demonstrated consistency in that the same ratios could identify the weathered oil to its corresponding unweathered source oil. Eight of the newly added APANH ratios had < 14% relative percent difference, indicating a confident match. Increased confidence was noted as these ratios showed a distinct match versus other unrelated source oils. These results therefore confirmed that the eight APANH diagnostic ratios determined in this study were consistently applicable in their enhancement of the CEN method.

### Enhancement to the CEN methodology

The eight selected APANH ratios were added to the CEN method in order to demonstrate the enhancement achieved. The previously noted visual assessment of this expanded method is complimented by review of the number of response ratios unflagged, i.e., within the < 14% criteria (Table 7). Overall, inclusion of these eight additional APANH diagnostic ratios showed < 14% relative percent difference for ≥ 59 out of the total 105 diagnostic ratios (Table 7; includes the CEN method). In comparison with other unweathered source oils, ratios indicated that the oils were not a match by significantly more ratios exceeding this value. The acceptance criterion for the expanded diagnostic ratio inclusive of PASHs and APANHs was therefore proposed to be a match when ≥ 59 out of the total 105 diagnostic ratios were < 14% relative difference.

With the newly proposed acceptance criteria, HFO TC oil by the classic CEN method has 38 (of 78) acceptable diagnostic ratio matches (Table 4). Inclusion of PASH and APASH ratios increased matches to 57 (of 99) (Table 5), and this was even further improved by the inclusion of the eight new APANH ratios, showing 65 (of 105) diagnostic ratios within acceptance criteria (Table 7). The overall result for the expanded diagnostic ratio method was a significant increase in the number of ratios that were unflagged, thus increasing confidence in the identification result. The results of this study demonstrate how the inclusion of stable APANH compounds as biomarkers strengthens diagnostic ratio analysis.

**Table 7:** Number of acceptable (unflagged; highlighted in italics) diagnostic ratios when comparing the weathered to unweathered oil samples by the classic CEN ratios plus additional PASH/APASH and proposed APANH ratios

Using classic biomarker ratios (78), PASH/APASH ratios (19), and APANH ratios (8)—total 105	HFO TC	PMRD	ASMB	HB	HFO 6303	MC
Weathered HFO TC	*65*	21	24	17	25	24
Weathered PMRD	24	*67*	31	21	20	29
Weathered ASMB	21	25	*66*	20	20	30
Weathered HB	11	12	16	*59*	14	30
Weathered HFO 6303	26	17	24	21	*71*	32
Weathered MC	17	23	27	29	24	*69*

**Fig. 4:**
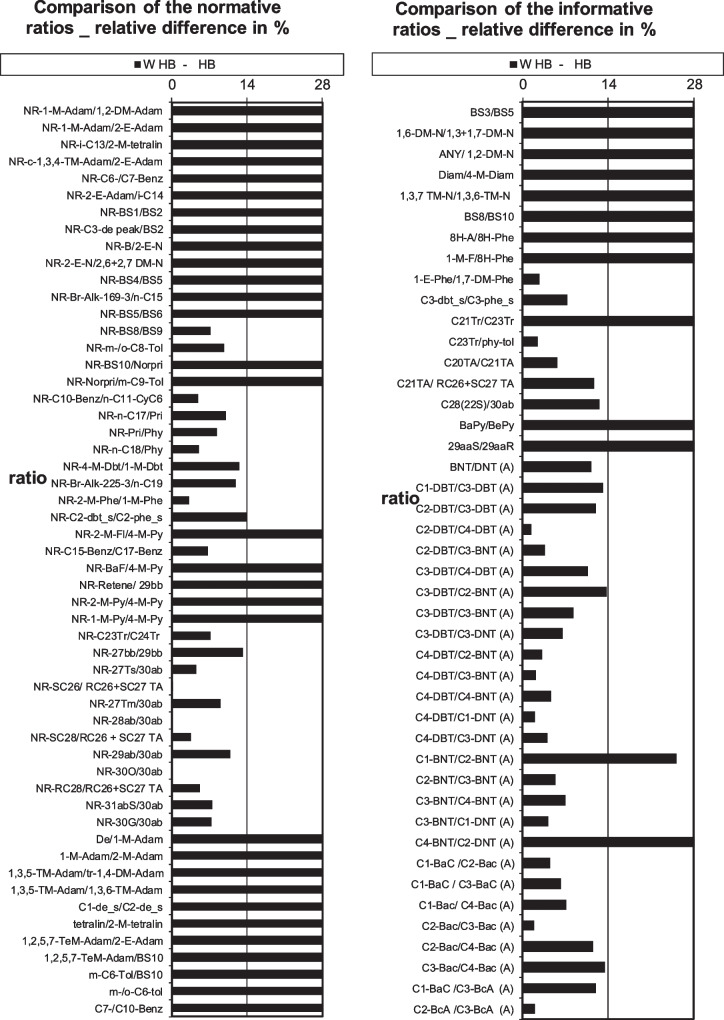
Visual representation of classic diagnostic ratio (78) results, PASH/APASH diagnostic ratio (+ 19) results, and APANH diagnostic ratio (+ 8) results included (right bottom) for the HB weathered versus corresponding source oil (FAME excluded for clarity of figure)

The increase in confidence once the additional APANH diagnostic ratios were included can be visually seen in Fig. 4. Additional visual charts for all of the tested petroleum oils can be found in SI Figs. S4–S8, demonstrating application of the final enhanced CEN method including PASHs, APASHs, and the new APANH diagnostic ratios. This visual representation is an asset in providing clear and understandable results to external personnel that do not possess the training to interpret more complex data. Matching spill sample to source sample using the presented procedure offers clarity and confidence in the result, a significant legal advantage when liability and damage is being assessed.

## Conclusion

Internationally accepted diagnostic ratio analysis is the main tool for determination of the source of a spilled oil by comparison with potential source oils. Recent publications enhanced the diagnostic ratio analysis though the inclusion of sulfur homologues, with the concept that additional non-classical biomarkers add to the fingerprint of an oil. In the current study, this concept was expanded further to screen for trace-level PANH and APANH compounds using the sensitivity and specificity of high-resolution GC-QToF analysis. High consideration was given to determine which of the nitrogen-containing compounds were able to survive extreme weathering conditions corresponding to local exposure in the Pacific Northwest marine waters. Those identified as being the most stable were six APANH compounds, i.e., C1-Benzo[a]carbazole, C2-Benzo[a]carbazole, C3-Benzo[a]carbazole, C4-Benzo[a]carbazole, C2-Benzo[c]acridines, and C3-Benzo[c]acridines. These biomarkers are four membered ring structures, resulting in their high stability during environmental weathering processes, with the inclusion of nitrogen adding variability to their classic APAH counterparts. The selected biomarkers formed the basis of eight ratios that were added to diagnostic ratio forensic analysis, enabling confident identification of oil spill source samples even post-extreme weathering of crude oil. Our study focused on a potential oil spill in a marine environment, while future research of these biomarkers will likely seek to determine their behavior in other matrixes, such as sediment and biota.

## Supplementary Information

Below is the link to the electronic supplementary material.

Supplementary file1 (DOCX 205 KB)

## Data Availability

Data will be provided upon request.
