# Supplementary material for: Enhancement of oil forensic methodology through the addition of polycyclic aromatic nitrogen heterocycle biomarkers for diagnostic ratios
Source: Environ Monit Assess. 2023 Feb 20;195(3):416. doi: 10.1007/s10661-023-10941-3 (PMC9941221; doi:10.1007/s10661-023-10941-3)
Supplement: Supplementary file 1 — Supplementary file1 (DOCX 205 KB) [file 10661_2023_10941_MOESM1_ESM.docx]

**Enhancement of Oil Forensic Methodology through the Addition of Polycyclic Aromatic Nitrogen Heterocycle Biomarkers for Diagnostic Ratios**

Paige McCallum, Taylor Filewood, Julia Sawitsky, Honoria Kwok, Pamela Brunswick*, Jeffrey Yan, Leah Chibwe, Krishnaja Tikkisetty, Dayue Shang*

Pacific Environmental Science Centre

Science and Technology Branch, Environment and Climate Change Canada

Vancouver, British Columbia, Canada

[Pamela.brunswick@ec.gc.ca](mailto:Pamela.brunswick@ec.gc.ca) and [Dayue.shang@ec.gc.ca](mailto:Dayue.shang@ec.gc.ca)

Reagents

The following reagents where purchased in 2021 and used for analysis in a study completed by [Filewood et al., 2022a; Filewood et al., 2022b; Filewood et al., 2022c]. From VWR (Mississauga, Canada) hexane and dichloromethane (DCM) were purchased of OmniSolv grade. From Fisher Scientific (Pittsburgh, PA, USA) anhydrous sodium sulphate, 10–60 mesh was purchased (Environmental Residue Analysis grade). Before use, silica gel (synthetic sodium silicate, anhydrous) was heat treated for 8 hours at 325 °C. To obtain ultra-high purity (UHP) water, a Millipore Milli-Q plus Ultra-Pure Water System was used. Seawater sourced from the Burrard Inlet (BC, Canada) was obtained using an on-site pumping system located at Pacific Environmental Science Centre (North Vancouver, Canada). The pump system was located at a depth of 33 m. The seawater had a salinity of 25.1 ppt, hardness 4060 mg/L CaCO3 and pH 7.72 before it underwent the weathering process.

Un-weathered oil extraction and sample preparation

The sample preparation procedure was reported previously [Filewood et al 2022b]. For each petroleum oil, a column clean-up using 10 mL Kimble Serological Pipette (borosilicate glass) columns loaded with silica gel, was carried out. Approximately 0.3 g of sodium sulfate was placed on top of the silica gel phase. The column was washed twice with DCM (10 mL) before adding 100 μL of the petroleum oil sample. A 15 mL glass vial was placed under each column to collect the eluting sample. DCM was added to each column until the vials were filled. Following a nitrogen blow down, a final volume of 10 mL was achieved for each sample. Approximately 1 - 2 mL of each sample was transferred to a gas chromatography (GC) vial for testing on the GC-QToF.

Microcosm weathering conditions

Petroleum oils underwent artificial weathering on-site at the Pacific Environmental Science Centre in North Vancouver, BC (N-43.3080. W-123.0014). The microcosm weathering experiment occurred between May 27th and July 23rd, 2021. The microcosm experiment was reported previously [Filewood et al 2022b]. A solution of 0.5 mL petroleum oil, 5 mL of hexane and 250 mL of seawater was added to 600 mL beakers. The beakers were placed in transparent aquarium tanks (26×32×51 cm (W x H x L)). To prevent wildlife from interfering with the microcosms, the lids were lifted slightly at 5 cm, allowing for adequate airflow. To induce different types of weathering, including photo-oxidation, biodegradation, and evaporation, the experiment was set up outdoors in combined direct and indirect sunlight depending upon time of day. The microcosm weathering experiment was carried out between May 27th and July 23rd, 2021, wherein the lowest temperature was recorded at 8 ℃ and the highest 32 °C [Past Weather in North Vancouver, British Columbia, Canada, 2021]. Abnormally high temperatures for this region resulted in the loss of water in the microcosm. As a result, UHP water was periodically added to the beakers to prevent changes in salinity and the oil samples from running dry. Further details are in SI Table S2 [Filewood et al., 2022b].

Weathered oil extraction and sample preparation

After approximately two months of artificial weathering, the weathered oil samples were decanted into 80 mL screw-top solvent bottles (VWR) [Filewood et al., 2022a; Filewood et al., 2022b; Filewood et al., 2022c]. Transferring of the weathered samples was carried out through the use of a binary solvent (DCM/hexane, v/v, 40/60) to fill the beakers and ensure any residue was not lost during sample transfer. The dissolved weathered oil samples had a final volume of 25 mL, made up using the same binary solvent. After one minute of vigorous shaking, UHP water was added to induce phase separation. During clean up, about 5mL of the top oil layer was loaded onto silica gel columns as detailed in the un-weathered oil extraction and sample preparation section.

GC-QToF procedure and data collection

The parameters chosen for the GC-QToF were based on previous work of authors’ in this laboratory [Chua et al., 2020a; Chua et al., 2021; Filewood et al., 2022a; Filewood et al., 2022b; Filewood et al., 2022c]. For the GC-QToF, an Agilent 7890B GC with an Agilent 7693 injector, in tandem with a 7250 quadrupole time of flight mass detector, was used for analysis. GC separation was performed on an Agilent DB-5MS+DG Integra-guard column (30 m x 0.25 mm id x 0.25 µm film thickness). The analysis was run with an injection volume of 1 µL in pulsed splitless mode with an inlet temperature and GC-MS transfer line temperature of 280 °C. The temperature programming began at 50 °C, held for 2 min, followed by an increase of 6 °C/min until a temperature of 310 °C was reached and held for 15 min. The helium carrier gas flow rate was set at 1.0 mL/min. For GC-QToF analysis, total ion chromatogram (TIC) scan mode was selected with MS mass cutoff of 50 m/z and a ToF mass range of 50 to 500 m/z. MS acquisition was in electron impact ionization positive (EI+) mode. The source and quadrupole temperature were 250 °C and 150 °C, respectively, and helium quench gas was added at 4.0 mL/min. The total run time was 60.333 minutes.

**Tables:**

| Abbreviation | Full Petroleum Oil Name | Oil Type | Location Collected | Year Collected | Source of Spill | Year of Spill |
| --- | --- | --- | --- | --- | --- | --- |
| HFO TC | Transport Canada – Oceanic HFO filter 09.02.30 | Heavy Fuel Oil | Oceanic | 2002/09/30 | n/a | n/a |
| PM RDR | Plains midstream Red Deer River, 08Jun2012 | Light Sour Crude Oil | West Bank of Red Deer River, Alberta, Canada | 2012/06/08 | Burst Pipeline | 2012/06/07 |
| ASMB 5 | Alberta Sweet Mixed Blend #5 | Crude | Alberta Canada | n/a | n/a | n/a |
| HB | Hibernia | Light Sweet Crude Oil | Hibernia Oil Field, Newfoundland Canada | n/a | Marine Oil Field | n/a |
| HFO 6303 | HFO 6303, ESTS 20051031-0601.2 BPH 22Nov2012 | Heavy Fuel Oil (Bunker C) | Imperial Oil Ltd., Nova Scotia, Canada | 2012/11/22 | n/a | n/a |
| MC 252 | MC-252 ESTS 20100916-1622.6 BPH 22Nov2012 | Riser Oil | Gulf of Mexico, Transocean's Deepwater Horizon Oil Rig | 2012/11/22 | Marine Oil Rig | December 2010 |

**Table S1** Specific details for each petroleum oil investigated.

| **Date** | **Amount UHP water added (mL)** | **Final volume (mL)** |
| --- | --- | --- |
| June 14^th^ 2021 | ~50 | 250 |
| June 28^th^ 2021 | ~200 | 250 |
| July 5^th^ 2021 | ~150 | 250 |
| July 14^th^ 2021 | ~100 | 250 |

**Table S2** Details on water level adjustments for six petroleum oils during the microcosm weathering experiment.

| **Compound Abbreviation** | **Compound Name** | **Compound Group** | **Target Ion** |
| --- | --- | --- | --- |
| 1_1-M-Adam (H) | 1-methyl-adamantane | adamantanes | 135.119 |
| 2_1,2-DM-Adam (H) | 1,2-dimethyl-adamantane | adamantanes | 149.1271 |
| 3_i-C13 (H) | 2,6-dimethylundecane | isoprenoids | 113.1326 |
| 4_2-M-Tetralin (H) | 2-methyl-1,2,3,4-tetrahydronaphtalene | hydro polycyclic aromatic hydrocarbons | 104.0624 |
| 5_c-1,3,4-TM-Adam (H) | cis-1,3,4-trimethyl-adamantane | adamantanes | 163.1471 |
| 6_C6_Benz (H) | hexyl-benzene | alkylbenzenes | 92.0621 |
| 7_i-C14 (H) | 2,6,10-trimethylundecane | isoprenoids | 113.1325 |
| 8_2-E-Adam (H) | 2-ethyl-adamantane | adamantanes | 135.119 |
| 9_BS1 (H) | bicyclic sesquiterpane 1 | sesquiterpanes | 179.1793 |
| 10_C3-de peak (H) | C3-decalin range peak (ratio with BS2) | decalines | 180.0939 |
| 11_BS2 (H) | bicyclic sesquiterpane 2 | sesquiterpanes | 179.1792 |
| 12_C7_Benz (H) | heptyl-benzene | alkylbenzenes | 92.0621 |
| 13_B (H) | biphenyl | polycyclic aromatic hydrocarbons | 154.0773 |
| 14_2-E-N (H) | 2-ethyl-naphthalene | alkyl polycyclic aromatic hydrocarbons | 156.0932 |
| 15_2,6+2,7-DM-N (H) | 2,6+2,7-dimethyl-naphthalene | alkyl polycyclic aromatic hydrocarbons | 156.0929 |
| 16_Br-Alk 169-3 (H) | Branched alkane m/z 169-3 | branched alkanes | 169.1935 |
| 17_BS4 (H) | bicyclic sesquiterpane 4 | sesquiterpanes | 123.1162 |
| 18_BS5 (H) | bicyclic sesquiterpane 5 | sesquiterpanes | 123.1162 |
| 19_BS6 (H) | bicyclic sesquiterpane 6 | sesquiterpanes | 123.1162 |
| 20_n-C15 (H) | pentadecane | isoprenoids | 169.1935 |
| 21_BS8 (H) | bicyclic sesquiterpane 8 | sesquiterpanes | 193.1948 |
| 22_BS9 (H) | bicyclic sesquiterpane 9 | sesquiterpanes | 193.1942 |
| 23_m-C8-Tol (H) | meta-octyl-toluene | alkyltoluenes | 106.0776 |
| 24_BS10 (H) | bicyclic sesquiterpane 10 | sesquiterpanes | 123.1162 |
| 25_o-C8-Tol (H) | ortho-octyl-toluene | alkyltoluenes | 106.0774 |
| 26_Norpri (H) | Norpristan = 2,6,10-trimethylpentadecane | isoprenoids | 113.1325 |
| 27_m-C9-Tol (H) | meta-nonyl-toluene | alkyltoluenes | 106.0776 |
| 28_C10_Benz (H) | decyl-benzene | alkylbenzenes | 92.0621 |
| 29_n-C17 (H) | heptadecane | isoprenoids | 85.1025 |
| 30_Pri (H) | i-C19 = Pristane= 2,6,10,14-tetramethylpentadecane | isoprenoids | 85.1008 |
| 31_n-C11-CyC6 (H) | n-undecyl cyclohexane | n-alkylcyclohexanes | 83.0852 |
| 32_n-C18 (H) | octadecane | isoprenoids | 85.1025 |
| 33_Phy (H) | i-C20 = Phytane = 2,6,10,14 tetramethylhexadecane | isoprenoids | 85.1008 |
| 34_4-M-Dbt (H) | 4-methyl-dibenzothiophene | sulfur polycyclic aromatic hydrocarbons | 198.0485 |
| 35_Br-Alk 225-3 (H) | Branched alkane 225-3 | branched alkanes | 225.2588 |
| 36_n-C19 (H) | nonadecane | isoprenoids | 225.2588 |
| 37_1-M-Dbt (H) | 1-methyl-dibenzothiophene | sulfur polycyclic aromatic hydrocarbons | 198.0482 |
| 38_2-M-Phe (H) | 2-methyl-phenanthrene | alkyl polycyclic aromatic hydrocarbons | 192.0924 |
| 39_FAME 16:0 (H) | FAME 16:0 | Fame | 74.036 |
| 40_1-M-Phe (H) | 1-methyl-phenanthrene | alkyl polycyclic aromatic hydrocarbons | 192.0923 |
| 41_C2-dbt_s (A) | C2-dibenzothiophenes | sulfur polycyclic aromatic hydrocarbons | 212.0649 |
| 42_C2-phe_s (A) | C2-phenanthrenes anthracenes | alkyl polycyclic aromatic hydrocarbons | 206.1083 |
| 43_FAME 18:0 (H) | FAME 18:0 | Fame | 74.036 |
| 44_2-M-Fl (H) | 2-methyl-fluoranthene | alkyl polycyclic aromatic hydrocarbons | 216.0928 |
| 45_C15-Benz (H) | pentadecyl-enzene | alkylbenzenes | 92.0621 |
| 46_BaF (H) | benzo(a)-fluorene | polycyclic aromatic hydrocarbons | 216.0921 |
| 47_Retene (H) | 1-methyl-7-isopropyl-phenantrene | alkyl polycyclic aromatic hydrocarbons | 219.1179 |
| 48_2-M-Py (H) | 2-methyl-pyrene | alkyl polycyclic aromatic hydrocarbons | 216.0928 |
| 49_4-M-Py (H) | 4-methyl-pyrene | alkyl polycyclic aromatic hydrocarbons | 216.0924 |
| 50_1-M-Py (H) | 1-methyl-pyrene | alkyl polycyclic aromatic hydrocarbons | 216.0927 |
| 51_C23Tr (H) | C23 tricyclic terpane | triterpanes | 191.1733 |
| 52_C24Tr (H) | C24 tricyclic terpane | triterpanes | 191.1783 |
| 53_C17-Benz (H) | heptadecyl-benzene | alkylbenzenes | 92.0621 |
| 54_27bbR+S (H) | 5α (H),14β(H),17β(H), 20(R+S)-cholestane | steranes | 218.2015 |
| 55_27Ts (H) | 18α(H)-22,29,30-trisnorneohopane | hopanes | 191.1776 |
| 56_SC26 TA (H) | C26,20S-triaromatic sterane | aromatic steranes | 231.1159 |
| 57_27Tm (H) | 17α(H)-22,29,30-trisnorhopane | hopanes | 191.1794 |
| 58_RC26TA+SC27 TA (H) | C26,20R- + C27,20S-triaromatic sterane | aromatic steranes | 231.1159 |
| 59_29bbR+S (H) | 24-ethyl-5α(H),14β (H),17β(H), 20(R+S)- cholestane | steranes | 218.2015 |
| 60_28ab (H) | 17α(H), 21β(H)-28,30-bisnorhopane | hopanes | 191.1783 |
| 61_SC28 TA (H) | C28,20S-triaromatic sterane | aromatic steranes | 231.1159 |
| 62_29ab (H) | 17α(H), 21β(H)-30-norhopane | hopanes | 191.1849 |
| 63_30O (H) | 18α(H)-oleanane | hopanes | 191.1758 |
| 64_30ab (H) | 17α(H), 21β(H)-hopane (hopane) | hopanes | 191.1751 |
| 65_RC28 TA (H) | C28,20R-triaromatic sterane | aromatic steranes | 231.1159 |
| 66_31abS (H) | 17α(H), 21β(H), 22(S)-homohopane | hopanes | 191.1788 |
| 67_30G (H) | gammacerane | hopanes | 191.1778 |
| 68_De (H) | Decalin | decalines | 138.1408 |
| 69_1,3,5-TM-Adam (H) | 1,3,5-trimethyl adamantane | adamantanes | 163.1465 |
| 70_C1-de_s (A) | C1-decalins | decalines | 152.1556 |
| 71_2-M-Adam (H) | 2-methyl adamantane | adamantanes | 135.119 |
| 72_Tetralin (H) | 1,2,3,4 tetrahydronaphtalene | hydro polycyclic aromatic hydrocarbons | 104.0625 |
| 73_c-1,4-DM-Adam (H) | cis-1,4-dimethyl adamantane | adamantanes | 149.1271 |
| 74_N (H) | naphthalene | polycyclic aromatic hydrocarbons | 128.0622 |
| 75_tr-1,4-DM-Adam (H) | trans-1,4-dimethyl adamantane | adamantanes | 149.1271 |
| 76_1,3,6-TM-Adam (H) | 1,3,6-trimethyl-adamantane | adamantanes | 163.1475 |
| 77_C2-de_s (A) | C2-decalins | decalines | 166.1703 |
| 78_tr-1,3,4-TM-Adam (H) | trans-1,3,4-trimethyl-adamantane | adamantanes | 163.1477 |
| 79_1,2,5,7-TeM-Adam (H) | 1,2,5,7-tetramethyl-adamantane | adamantanes | 177.1632 |
| 80_1-M-N (H) | 1-methyl-naphthalene | alkyl polycyclic aromatic hydrocarbons | 142.0772 |
| 81_m-C6-Tol (H) | meta-hexyl-toluene | alkyltoluenes | 106.0776 |
| 82_Br-Alk 169-1 (H) | Branched alkane 169-1 | branched alkanes | 169.1935 |
| 83_o-C6-Tol (H) | ortho-hexyl-toluene | alkyltoluenes | 106.0772 |
| 84_i-C15 (H) | Farnesane = 2,6,10 trimethyldodecane | isoprenoids | 113.1325 |
| 85_1,3+1,7-DM-N (H) | 1,3+1,7-dimethyl-naphthalene | alkyl polycyclic aromatic hydrocarbons | 156.093 |
| 86_1,6-DM-N (H) | 1,6-dimethyl-naphthalene | alkyl polycyclic aromatic hydrocarbons | 156.093 |
| 87_BS3 (H) | bicyclic sesquiterpane 3 | sesquiterpanes | 123.1162 |
| 88_C2-bt_s (A) | C2-benzothiophenes | sulfur polycyclic aromatic hydrocarbons | 162.0469 |
| 89_ANY (H) | acenaphthylene | polycyclic aromatic hydrocarbons | 152.0611 |
| 90_m-C7-Tol (H) | meta-heptyl-toluene | alkyltoluenes | 106.0776 |
| 91_Br-Alk 169-2 (H) | Branched alkane 169-2 | branched alkanes | 169.1935 |
| 92_1,2-DM-N (H) | 1,2 -dimethyl-naphthalene | alkyl polycyclic aromatic hydrocarbons | 156.0929 |
| 93_i-C16 (H) | 2,6,10 trimethyltridecane | isoprenoids | 113.1323 |
| 94_o-C7-Tol (H) | ortho-heptyl-toluene | alkyltoluenes | 106.0773 |
| 95_Diam (H) | diamantane | diamondoids | 188.1565 |
| 96_FAME 12:0 (H) | FAME 12:0 | Fame | 74.036 |
| 97_4-M-Diam (H) | 4-methyl-diamantane | diamondoids | 187.1492 |
| 98_1,3,7-TM-N (H) | 1,3,7-trimethyl-naphthalene | alkyl polycyclic aromatic hydrocarbons | 170.1089 |
| 99_1,3,6-TM-N (H) | 1,3,6-trimethyl-naphthalene | alkyl polycyclic aromatic hydrocarbons | 170.1083 |
| 100_n-C9-CyC6 (H) | n-nonyl cyclohexane | n-alkylcyclohexanes | 83.0852 |
| 101_o-C9-Tol (H) | ortho-nonyl-toluene | alkyltoluenes | 106.0775 |
| 102_8H-A (H) | 1,2,3,4,5,6,7,8-octahydroanthracene | hydro polycyclic aromatic hydrocarbons | 186.1409 |
| 103_1-M-F (H) | 1-methyl-fluorene | alkyl polycyclic aromatic hydrocarbons | 180.0825 |
| 104_8H-Phe (H) | 1,2,3,4,5,6,7,8_octahydrophenanthrene | hydro polycyclic aromatic hydrocarbons | 186.1409 |
| 105_FAME 14:0 (H) | FAME 14:0 | Fame | 74.036 |
| 106_Br-Alk 225-1 (H) | Branched alkane 225-1 | branched alkanes | 225.2588 |
| 107_C2-f_s (A) | C2-fluorenes | alkyl polycyclic aromatic hydrocarbons | 194.1084 |
| 108_Br-Alk 225-2 (H) | Branched alkane 225-2 | branched alkanes | 225.2588 |
| 109_m-C11-Tol (H) | meta-undecyl-toluene | alkyltoluenes | 106.0777 |
| 110_C12-Benz (H) | dodecyl-benzene | alkylbenzenes | 92.0621 |
| 111_o-C11-Tol (H) | ortho-undecyl-toluene | alkyltoluenes | 106.0774 |
| 112_FAME 16:1 (H) | FAME 16:1 | Fame | 74.036 |
| 113_2-M-A (H) | 2-methyl-anthracene | alkyl polycyclic aromatic hydrocarbons | 192.0926 |
| 114_1-E-Phe (H) | 1-ethyl-phenanthrene | alkyl polycyclic aromatic hydrocarbons | 206.1085 |
| 115_1,7 DM-Phe (H) | 1,7-dimethyl-phenanthrene | alkyl polycyclic aromatic hydrocarbons | 206.1082 |
| 116_C3-dbt_s (A) | C3-dibenzothiophenes | sulfur polycyclic aromatic hydrocarbons | 226.0796 |
| 117_FAME 18:2 (H) | FAME 18:2 | Fame | 74.036 |
| 118_FAME 18:1+18:3 (H) | FAME 18:1 + FAME 18:3 | Fame | 74.036 |
| 119_C21Tr (H) | C21 tricyclic terpane | triterpanes | 290.2979 |
| 120_C3-phe_s (A) | C3-phenanthrenes anthracenes | alkyl polycyclic aromatic hydrocarbons | 220.1243 |
| 121_C4-phe_s (A) | C4-phenanthrenes anthracenes | alkyl polycyclic aromatic hydrocarbons | 234.1414 |
| 122_FAME 20:1 (H) | FAME 20:1 | Fame | 74.036 |
| 123_Te-M-Phe (H) | tetramethyl-phenanthrene | alkyl polycyclic aromatic hydrocarbons | 234.1414 |
| 124_FAME 20:0 (H) | FAME 20:0 | Fame | 74.036 |
| 125_C2-fl (A) | C2-fluoranthrenes pyrenes | alkyl polycyclic aromatic hydrocarbons | 230.108 |
| 126_BNT (H) | Benzo[b]naphtho[1,2-d]thiophene | sulfur polycyclic aromatic hydrocarbons | 234.1414 |
| 127_C20TA (H) | C20-triaromatic sterane | aromatic steranes | 231.1159 |
| 128_C25Trab (H) | C25 tricyclic terpane (a+b) | triterpanes | 191.1812 |
| 129_C21 TA (H) | C21-triaromatic sterane | aromatic steranes | 231.1159 |
| 130_Phy-Tol (H) | phytanyl-toluene (1-methyl-3-phytanylbenzene) | alkyltoluenes | 106.0779 |
| 131_C1-chr (A) | C1-chrysenes | alkyl polycyclic aromatic hydrocarbons | 242.1088 |
| 132_27dbS (H) | 13β (H),17α(H), 20S - cholestane | steranes | 217.1947 |
| 133_27dbR (H) | 13β (H),17α(H), 20R - cholestane | steranes | 217.1943 |
| 134_C28 (22S) (H) | C28 tricyclic terpane | hopanes | 191.1738 |
| 135_C29 (22S) (H) | C29 tricyclic terpane | hopanes | 191.1755 |
| 136_BePy (H) | benzo (e) pyrene | polycyclic aromatic hydrocarbons | 252.0926 |
| 137_BaPy (H) | benzo (a) pyrene | polycyclic aromatic hydrocarbons | 252.0921 |
| 138_28bbR+S (H) | 24-methyl-5α(H),14β (H),17β(H), 20(R+S)-cholestane | steranes | 218.2003 |
| 139_28aaR (H) | 24-methyl-5α(H),14α(H),17α(H), 20R- cholestane | steranes | 217.1938 |
| 140_29aaS (H) | 24-ethyl-5α(H),14α(H),17α(H), 20S- cholestane | steranes | 217.1941 |
| 141_25nor30ab (H) | 17α(H), 21β(H)-25,28,30-trisnorhopane | hopanes | 177.163 |
| 142_29aaR (H) | 24-ethyl-5α(H),14α(H),17α(H), 20R- cholestane | steranes | 217.1027 |
| 143_29Ts (H) | 18α(H)-30-norneohopane | hopanes | 191.1761 |
| 144_30ba (H) | 17β(H), 21α(H)-hopane (moretane) | hopanes | 191.187 |
| 145_32abS (H) | 17α(H), 21β(H), 22S-bishomohopane | hopanes | 191.1796 |
| 146_BT | Benzothiophene | polycyclic aromatic sulfur heterocycle | 134.0185 |
| 147_DBT | Dibenzothiophene | polycyclic aromatic sulfur heterocycle | 184.0341 |
| 148_BNT | Benzonaphthiophene | polycyclic aromatic sulfur heterocycle | 234.0498 |
| 149_DNT | Dinaphthothiophene | polycyclic aromatic sulfur heterocycle | 284.0654 |
| 150_C1-DBT | C1-Dibenzothiophene | polycyclic aromatic sulfur heterocycle | 198.0498 |
| 151_C2-DBT | C2-Dibenzothiophene | polycyclic aromatic sulfur heterocycle | 212.0654 |
| 152_C3-DBT | C3-Dibenzothiophene | polycyclic aromatic sulfur heterocycle | 226.0811 |
| 153_C4-DBT | C4-Dibenzothiophene | polycyclic aromatic sulfur heterocycle | 240.0967 |
| 154_C1-BNT | C1-Benzonaphthothiophene | polycyclic aromatic sulfur heterocycle | 248.0654 |
| 155_C2-BNT | C2-Benzonaphthothiophene | polycyclic aromatic sulfur heterocycle | 262.0811 |
| 156_C3-BNT | C3-Benzonaphthothiophene | polycyclic aromatic sulfur heterocycle | 276.0967 |
| 157_C4-BNT | C4-Benzonaphthothiophene | polycyclic aromatic sulfur heterocycle | 290.1124 |
| 158_C1-DNT | C1-Dinapthothiophene | polycyclic aromatic sulfur heterocycle | 298.0811 |
| 159_C2-DNT | C2-Dinapthothiophene | polycyclic aromatic sulfur heterocycle | 312.0967 |
| 160-C3-DNT | C3-Dinapthothiophene | polycyclic aromatic sulfur heterocycle | 326.1124 |
| 161-C4-DNT | C4-Dinapthothiophene | polycyclic aromatic sulfur heterocycle | 340.128 |
| 162_C1-BaC | C1-Benzo[a]carbazole | polycyclic aromatic nitrogen heterocycle | 231.1048 |
| 163_C2-BcA | C2-Benzo[c]acridines | polycyclic aromatic nitrogen heterocycle | 257.1204 |
| 164_C2-BaC | C2-Benzo[a]carbazole | polycyclic aromatic nitrogen heterocycle | 245.1204 |
| 165_C3-BcA | C3-Benzo[c]acridines | polycyclic aromatic nitrogen heterocycle | 271.1361 |
| 168_C3-BaC | C3-Benzo[a]carbazoles | polycyclic aromatic nitrogen heterocycle | 259.1361 |
| 169_C4-BaC | C4-Benzo[a]carbazoles | polycyclic aromatic nitrogen heterocycle | 273.1517 |

**Table S3** List of all compounds analyzed and their target ions.

| APANH diagnostic ratios | Area or height used |
| --- | --- |
| C2-Benzo[c]Acridines /C3-Benzo[c]Acridines | Area |
| C1-Benzo[a]Carbazole /C2-Benzo[c]Acridines | Area |
| C1-Benzo[a]Carbazole /C2-Benzo[a]Carbazole | Area |
| C1-Benzo[a]Carbazole /C3-Benzo[a]Carbazole | Area |
| C1-Benzo[a]Carbazole /C4- Benzo[a]Carbazole | Area |
| C2-Benzo[a]Carbazole /C3- Benzo[a]Carbazole | Area |
| C2-Benzo[a]Carbazole /C4- Benzo[a]Carbazole | Area |
| C3-Benzo[a]Carbazole /C4- Benzo[a]Carbazole | Area |

**Table S4** Compound area or height selected for diagnostic ratio determination.

**Figures:**


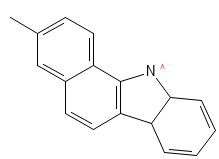

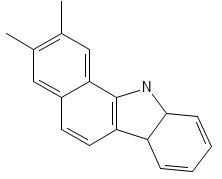

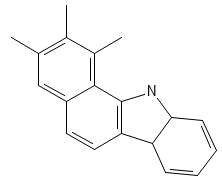


C1- Benzo[a]carbazoles C2-Benzo[a]carbazoles C3-Benzo[a]carbazoles


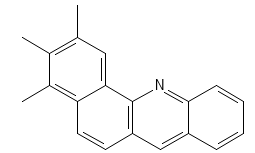


C4-Benzo[a]carbazoles C2-Benzo[c]acridines C3-Benzo[c]acridines

**Fig S1** Names and structures of the six determined biomarkers.

**Fig S2** Visual representation of classic diagnostic ratio (78) results, PASH/APASH diagnostic ratio (+19) results, and APANH diagnostic ratio (+8) results included (right bottom) for the duplicate HB weathered versus corresponding source oil (FAME excluded for clarity of figure).

**Fig S3** Visual representation of classic diagnostic ratio (78) results, PASH/APASH diagnostic ratio (+19) results, and APANH diagnostic ratio (+8) results included (right bottom) for the duplicate MC 252 weathered versus corresponding source oil (FAME excluded for clarity of figure).

**Fig S4** Visual representation of classic diagnostic ratio (78) results, PASH/APASH diagnostic ratio (+19) results, and APANH diagnostic ratio (+8) results included (right bottom) for the PM RDR weathered versus corresponding source oil (FAME excluded for clarity of figure).

**Fig S5** Visual representation of classic diagnostic ratio (78) results, PASH/APASH diagnostic ratio (+19) results, and APANH diagnostic ratio (+8) results included (right bottom) for the MC 252 weathered versus corresponding source oil (FAME excluded for clarity of figure).

**Fig S6** Visual representation of classic diagnostic ratio (78) results, PASH/APASH diagnostic ratio (+19) results, and APANH diagnostic ratio (+8) results included (right bottom) for the ASMB 5 weathered versus corresponding source oil (FAME excluded for clarity of figure).

**Fig S7** Visual representation of classic diagnostic ratio (78) results, PASH/APASH diagnostic ratio (+19) results, and APANH diagnostic ratio (+8) results included (right bottom) for the HFO TC weathered versus corresponding source oil (FAME excluded for clarity of figure).

**Fig S8** Visual representation of classic diagnostic ratio (78) results, PASH/APASH diagnostic ratio (+19) results, and APANH diagnostic ratio (+8) results included (right bottom) for the HFO 6303 weathered versus corresponding source oil (FAME excluded for clarity of figure).
